# Specific Depletion of Leukemic Stem Cells: Can MicroRNAs Make the Difference?

**DOI:** 10.3390/cancers9070074

**Published:** 2017-06-30

**Authors:** Tania Martiáñez Canales, David C. de Leeuw, Eline Vermue, Gert J. Ossenkoppele, Linda Smit

**Affiliations:** Department of Hematology, VU University Medical Center, Cancer Center Amsterdam, Boelelaan 1117, 1081 HV Amsterdam, The Netherlands; t.martianezcanales@vumc.nl (T.M.C.); d.deleeuw@vumc.nl (D.C.d.L.); e.vermue@vumc.nl (E.V.); G.Ossenkoppele@vumc.nl (G.J.O.)

**Keywords:** MicroRNAs, AML, leukemic stem cells, hematopoietic stem cells

## Abstract

For over 40 years the standard treatment for acute myeloid leukemia (AML) patients has been a combination of chemotherapy consisting of cytarabine and an anthracycline such as daunorubicin. This standard treatment results in complete remission (CR) in the majority of AML patients. However, despite these high CR rates, only 30–40% (<60 years) and 10–20% (>60 years) of patients survive five years after diagnosis. The main cause of this treatment failure is insufficient eradication of a subpopulation of chemotherapy resistant leukemic cells with stem cell-like properties, often referred to as “leukemic stem cells” (LSCs). LSCs co-exist in the bone marrow of the AML patient with residual healthy hematopoietic stem cells (HSCs), which are needed to reconstitute the blood after therapy. To prevent relapse, development of additional therapies targeting LSCs, while sparing HSCs, is essential. As LSCs are rare, heterogeneous and dynamic, these cells are extremely difficult to target by single gene therapies. Modulation of miRNAs and consequently the regulation of hundreds of their targets may be the key to successful elimination of resistant LSCs, either by inducing apoptosis or by sensitizing them for chemotherapy. To address the need for specific targeting of LSCs, miRNA expression patterns in highly enriched HSCs, LSCs, and leukemic progenitors, all derived from the same patients’ bone marrow, were determined and differentially expressed miRNAs between LSCs and HSCs and between LSCs and leukemic progenitors were identified. Several of these miRNAs are specifically expressed in LSCs and/or HSCs and associated with AML prognosis and treatment outcome. In this review, we will focus on the expression and function of miRNAs expressed in normal and leukemic stem cells that are residing within the AML bone marrow. Moreover, we will review their possible prospective as specific targets for anti-LSC therapy.

## 1. Introduction

The treatment outcome of acute myeloid leukemia (AML) patients depends on several factors, including karyotype and molecular alterations present in the leukemic cell bulk. Combination chemotherapy leads to complete remission (CR) in the majority of patients [1]. However, 50% of patients that have been in CR develop a relapse within 5 years after their initial diagnosis. This recurrence of the disease is thought to be caused by chemotherapy resistant leukemic cells with stem cell-like properties, named “leukemic stem cells” (LSCs) [2,3,4]. To improve the treatment outcome of AML patients it will be crucial to eradicate LSCs to finally prevent relapse. LSCs are functionally defined by their ability to initiate AML in immunodeficient mice [5], and were initially identified as a population of leukemic cells with a CD34+CD38− immunophenotype, similar to normal hematopoietic stem cells (HSCs) [4,5]. However, LSCs showed to be more heterogeneous than the CD34+CD38− phenotype and to reside also in other cell compartments [6,7,8,9]. Moreover, at AML relapse, LSC frequency and phenotypic diversity showed to much greater than at diagnosis, indicating that chemotherapy promotes changes in the LSC compartment [10]. In contrast to what is observed in the patient at relapse, cytarabine resistant cells generated in an AML xenograft mouse model are not enriched for the CD34+CD38− phenotype or for cells containing enhanced functional leukemia-initiating potential, neither were these cells enriched for stem cell genes [8]. The clinical importance of LSCs was shown by a study of Ng et al., in where it was demonstrated that the presence of a 17 gene LSC expression signature derived from functionally defined LSCs could predict the risk for relapse [11]. Altogether, to improve treatment outcome for AML patients it will be crucial to eradicate the dynamic LSC compartment during the disease course.

**Figure 1:**
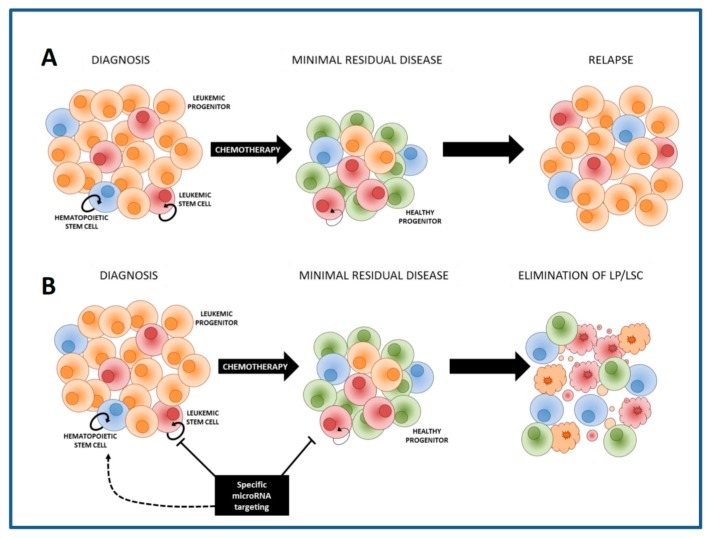
Role of LSCs in relapse development. At diagnosis, AML consist of a heterogeneous population of leukemic (stem) cells and residual normal hematopoietic (stem) cells. (**A**) Treatment with chemotherapy often results in complete remission. However, small numbers of leukemic cells survive the treatment (minimal residual disease, MRD). MRD contains chemotherapy resistant LSCs, which have the capacity to re-initiate leukemia and form a relapse. (**B**) MicroRNA-based therapy in combination with chemotherapy could eradicate LSCs and leukemic progenitors (LP) while sparing, or stimulating, HSCs.

LSCs co-exist with residual normal CD34+CD38− HSCs in the bone marrow of the AML patient. Increasing the chemotherapy dose might eliminate LSCs, nevertheless will inevitably result in the non-specific elimination of HSCs, leading to prolonged or permanent marrow aplasia and other toxicities. Therefore, it will be crucial to develop additional therapies that specifically eradicate LSCs but that will spare HSCs (Figure 1). Several cell properties enabling discrimination of LSCs from HSCs within AML bone marrows were identified, including expression of CLEC12A (CLL-1), CD123, TIM-3, CD34 and CD45, scatter properties and activity of aldehyde dehydrogenases [12,13,14,15,16].

Previously, gene expression profiling (GEP) has been performed to uncover transcriptional programs present in normal bone marrow and AML cells and to discriminate between AML subtypes and responding and non-responding patients. Moreover, the presence of a gene expression profile GEP can also predict patient survival [5,6,7,8]. However, GEP has not often succeeded in the uncovering of genes that upon targeting specifically eradicate leukemia cells, leaving the HSCs untouched. The reason for this might be that a complex phenomenon like therapy resistance and cancer cell maintenance is not easily overruled by targeting a single gene. To unravel chemotherapy resistance mechanisms present in LSCs, several studies compared the gene expression signature of CD34+CD38− LSCs or functionally defined LSCs with that of non-LSC compartments, AML blasts or leukemic progenitors [11,17,18,19]. To identify whether potential anti-LSC targets might be specific and sparing healthy HSCs, GEPs of LSCs were compared with those of HSCs [20,21,22]. All these past studies compared the gene expression of AML LSCs with that of HSCs derived from healthy donors. However, as AML induce changes in HSCs and in the healthy bone marrow microenvironment, thereby suppressing HSC function and supporting LSC survival, self-renewal and chemotherapy resistance [23,24,25,26] these studies did not take into account the changes induced in HSCs by the leukemia itself. As LSCs utilize a variety of mechanisms to resist chemotherapy and to drive relapse, the major challenge in targeting all the leukemic cells that contain stem cell properties is their heterogeneity. MiRNA-based therapeutic strategies might successfully eliminate a large population of AML cells with stem cell features as they target hundreds of genes at the same time.

## 2. MicroRNAs

**Figure 2:**
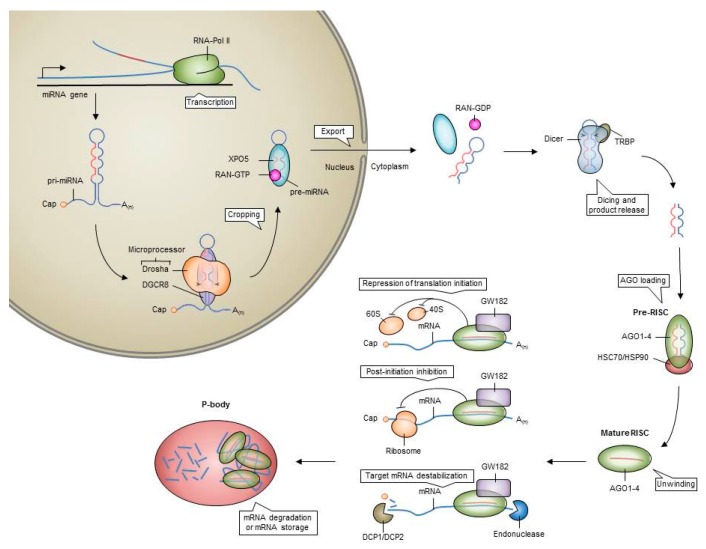
MicroRNA biogenesis and function. Primary miRNAs (pri-miRNA) are transcribed from miRNA genes by RNA polymerase II (RNA-Pol II). In the nucleus, the endonuclease Drosha together with a double-stranded RNA (dsRNA)-binding protein (DGCR8) cleave the stem–loop structure of the pri-miRNA. This results in a precursor miRNA (pre-miRNA) which is exported from the nucleus by exportin 5 (XPO5). In the cytoplasm, the pre-miRNA is then further cleaved by the endonuclease Dicer leading to a miRNA–miRNA* duplex. This duplex is loaded into an Argonaute (AGO) protein. The mature RNA-induced-silencing complex (RISC) is formed when miRNA*-strand is expelled from the AGO protein. The RISC complex can inhibit initiation of translation by affecting recruitment of 40S small ribosomal subunit and/or by inhibiting the 60S subunit. Alternatively, RISC may obstruct translation by inhibiting the elongation of ribosomes. RISC binding can also lead to recruitment of RNA decapping and/or deadenylating enzymes leading to mRNA destabilization. Some of the target mRNAs bound by the RISC are transported into cytoplasmic processing bodies (P-bodies) for degradation or storage.

MicroRNAs (MiRNAs) are a class of small, non-coding RNAs of 18–25 nucleotides that post-transcriptionally control the translation and stability of mRNAs [27]. A miRNA is synthesized as a long RNA transcript known as pri-miRNA, which is cleaved by the RNAse III endoribonuclease Drosha to a pre-miRNA. This pre-miRNA is further processed in the cytoplasm by the protein Dicer to a mature functional miRNA [27]. The mature miRNA is silencing its target genes via mRNA degradation or via prevention of translation of the mRNA (Figure 2). By targeting tens to hundreds of genes at the same time, miRNAs can control basic biological functions and pathways such as HSC differentiation. Distinct miRNAs fine-tune each step of haematopoiesis, including the number and repopulation potential of HSCs [28,29,30]. Dicer showed to be essential for the persistence of HSCs and specifically miR-125a is able to control the number of HSCs by regulating hematopoietic stem/progenitor cell apoptosis [31] and long-term repopulating stem cell potential of mouse and human progenitors [30]. Deregulation of miRNA expression and function in hematopoietic cells can result in the development of cancer and cancer progression [32,33].

AML is characterized by founder mutations in an HSC or a more differentiated progenitor, disrupting the differentiation pathway originating from these cell populations and leading to abnormal miRNA expression patterns. A aberrant miRNA signatures strongly correlate with tumour classification, cytogenetics, molecular abnormalities, prognosis and therapy response [34,35,36]. Strikingly, miRNA expression profiles can provide prognostic information that is complementing cytogenetics, mutation analysis, and gene expression data [34] and can even be more potent in disease classification and prediction of therapy response and outcome than a GEP. For example, a miRNA expression profile could better classify acute leukemia of ambiguous lineage as either lymphoid or myeloid [37] than a GEP [38]. The outperformance of miRNA GEPs as compared to mRNA GEPs might be due to the enhanced stability of miRNAs as compared to mRNAs. Recently, it was also shown that specific serum exosome miRNAs can function as biomarkers for AML, circumventing the use of invasive bone-marrow aspirations and dependence on available leukemic blasts [39].

The aberrant expression of miRNAs contributes to the character of the tumor and can be either oncogenic or tumor suppressive depending on the cellular context and available expressed miRNA targets. In view of heterogeneity of LSCs and AML as being a heterogeneous disease, not successfully treated by targeting a single gene, miRNA modulation may hold the key to successful elimination of therapy resistant leukemic (stem) cells either by inducing apoptosis or by sensitizing for chemotherapy. Moreover, since there is differential expression of miRNAs in AML and normal bone marrow as well as in LSCs and HSCs and LSCs and leukemic progenitors, agents that modulate activity might potentially lead to leukemia and/or LSC-specific effects.

By using aldehyde dehydrogenases (ALDH) activity miRNA expression profiles of LSCs, leukemic progenitors and HSCs all obtained from the same AML sample were obtained [16,40]. This approach resulted in identification of miRNAs differentially expressed between LSC and HSC populations and between LSCs and leukemic progenitors [40]. Several of those miRNAs showed to be specifically expressed in LSCs and/or HSCs, and to have prognostic value in AML and/or to function as targets for miRNA-based anti-LSC therapy [40,41].

## 3. MicroRNAs in Healthy HSCs

HSCs can undergo self-renewal and give rise to all the cells of the hematopoietic system during life. To do so, there is a balance between self-renewal and differentiation that is strictly controlled by several molecular factors, including the activity of miRNAs. Over the last years, the expression and function of miRNAs during hematopoiesis have been intensively studied however studies about the expression of miRNAs in highly enriched human stem and progenitor cell populations are scarce. This is partly due to a lack of consensus on which markers to use for isolation of pure HSCs but also due to the difficulty in isolating sufficient numbers of HSCs for profiling. In general, human HSCs reside within the immuno-phenotypical defined compartment of lineage negative (Lin−) CD34+CD38−CD90+CD49f+CD45RA− cells, which differentiate into Lin−CD34+CD38−CD90−CD49f−CD45RA−(Thy−1^neg-lo^) [42] multipotent progenitors (MPP) containing both lymphoid and myeloid potential [43,44] The more committed Lin−CD34+CD38+ common myeloid progenitors (CMP), granulocyte-macrophage progenitors (GMP) and megakaryocyte-erythroid progenitors (MEP) that develop from the MPP, can be separated using differential expression of CD123, CD110 and CD45RA [45,46]. HSCs and immature progenitors are also characterized by expression of CD133 [47,48].

The expression of miRNAs has been mostly studied in fractions containing murine HSCs [49,50], in human CD34+ and CD133+ cell fractions [51,52,53,54] and in human CD34+CD38− [49] and CD90+CD45RA− fractions [55,56]. The group of Georgantas performed the first large scale miRNA profiling of human CD34+ peripheral blood and bone marrow cells and identified 33 miRNA [51]. Since then, other groups have performed similar analysis in more HSC-enriched fractions. Both miR-29a and miR-125a/b consistently showed higher expression in HSCs (Lin−CD34+CD38− CD90+CD45RA−) and multipotent progenitors (Lin−CD34+CD38−CD90−CD45RA−) than in committed and differentiated progenitors [55,56]. Depletion of miR-29a resulted in decreased numbers of HSCs and progenitors, decreased HSC self-renewal, increased HSC cell cycling and apoptosis, which is partly due to the enhanced expression of DNMT3A [57]. Ectopic expression of miR-29a in mouse HSC/progenitors resulted in acquisition of self-renewal capacity, a bias to myeloid differentiation, and induction of a myeloproliferative disorder that can develop into AML [55]. Comparing the expression of miRNAs in Lin−Sca−1+c−Kit+ (LSK), Lin−Sca−1−c−Kit+ (LS−K+), erythroid and myeloid mouse cells identified 131 miRNAs differentially expressed between these cell types. MiR-99b, let-7e and miR-125a showed to be highly expressed in LSKs and down-regulated upon differentiation. Overexpression of miR-125a increases the number of cobblestone-area forming cells and overexpression of miR-99b/let-7e/miR125a or miR-125a alone keeps the mouse HSCs in a primitive state [58] (Table 1). Ectopic expression of miR-125a in murine and human multipotent progenitors resulted also in increased self-renewal and robust long-term multi-lineage repopulation in transplanted recipient mice [30]. Besides enhancing HSC self-renewal potential, the size of the HSC population is modulated by miR-125a by regulating apoptosis [31] (Table 1).

An expression profiling and functional study by O’Connell et al identified 11 miRNAs enriched in HSCs. Ectopic expression of these miRNAs in normal bone marrow identified miR-125b as the miR that induces the greatest increase in repopulation potential [50]. Moreover, Ooi et al showed that miR-125b overexpression led to a reduction in apoptosis in HSCs [56]. Thus, miR-125b promotes self-renewal and inhibits apoptosis in HSCs [50,56,58].

MiR-126 and miR-130a are expressed in HSCs and early progenitors from both mice and human, but not in differentiated progenitors [59]. Downregulation of miR-126 in HSCs results in enhanced hematopoietic stem/progenitor cell proliferation without inducing exhaustion, resulting in expansion of mouse and human long-term repopulating HSCs. Decreased miR-126 increases also cell cycle progression and the number of HSCs (CD34+CD38-CD90+CD45RA−) [60].

Among the numerous miRNA expression studies in HSCs and progenitors there is considerable variation in results. This is partly due to the use of different profiling methods but also due to profiling of different immune-phenotypically defined HSCs that were derived from different sources; e.g., whole bone marrow, total CD34+ population or progenitor populations. Several studies investigating the functional role of specific miRNAs in normal hematopoiesis have been published and many of the identified miRNAs showed to affect progenitor lineage commitment and functions of mature hematopoietic cells (Table 1). Leukemia-inducing mutations cause aberrant miRNA expression in HSCs and or progenitors, resulting in impaired differentiation, apoptosis and/or self-renewal [128].

## 4. Differential Expression of MicroRNAs between LSCs and HSCs Residing within the AML Bone Marrow

The CD34+CD38− cell compartment residing within the AML bone marrow includes both leukemic and normal stem cells [4]. Both stem cell compartments have many features in common and the extent to which they differ is important for development of therapies targeting relapse-initiating cells while sparing HSCs. The properties of normal HSCs are influenced by the leukemic microenvironment but also by the AML cells themselves [24,25,26]. It has even been shown that AML cells can suppress hematopoiesis by the miRNAs in exosomes released from the AML cells [24]. Since LSCs and HSCs are influenced by both the leukemic microenvironment and the leukemic cells, searching for differences in miRNA expression between HSCs and LSCs that are both obtained from the AML patient’s bone marrow will enhance the chance of finding genuine anti-LSC targets.

**Figure 3:**
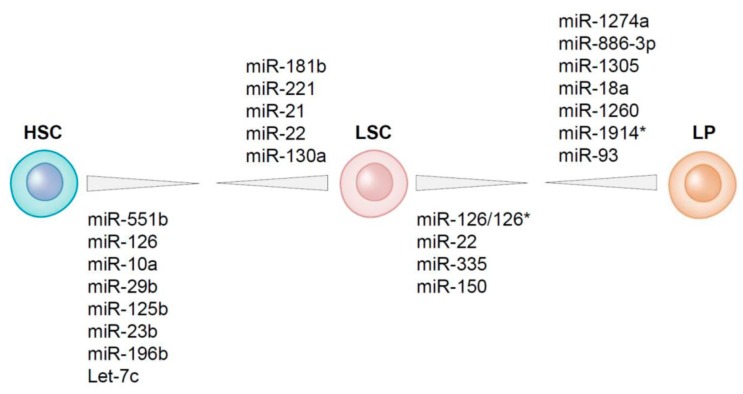
Differential expressed miRNAs between normal and leukemic stem cells and leukemic stem and progenitor cells obtained from AML bone marrow.

For the purification of HSCs and LSCs from an AML bone marrow the unequivocal separation of both compartments is necessary. LSCs often have aberrant protein expression, i.e., cell surface markers that do not fit to their lineage or maturation state [12,14,15]. Frequently observed non-myeloid lineage markers that are often used to distinguish between leukemic and normal myeloid cells are for example CD7, CD19, CD11b and CD56 [129]. These lineage markers are generally absent on normal HSCs while expressed in a subset of AML cases on leukemic stem and progenitor cells [14]. Other markers that are specifically expressed on LSCs and lacking on HSCs are for example CLL-1 and CD123 [15,130]. Generally, the expression of an aberrant immune-phenotypic marker is not absolute; i.e., not expressed on all leukemic cells within one patient, but also not present in all AML patients, which makes it difficult to use one particular biomarker for the isolation of LSCs and HSCs from all the AML patients [reviewed in [131]. Since HSCs have high ALDH activity and CD34+CD38− LSCs are in general characterized by lower ALDH activity, this problem can be circumvented by using ALDH activity as a functional biomarker. Using the difference in ALDH activity in combination with detection of aberrant leukemia-associated marker expression, CD34+CD38− HSCs and CD34+CD38− LSCs from AML bone marrows have been purified. After determining the expression of miRNAs in these cell fractions the comparison of the miRNA profiles of LSCs with those of residual HSCs revealed that MiR-551b, miR-10a, miR-151-5p, miR-29b and miR-125b are higher expressed in HSCs than in LSCs while miR-181b, miR-221, miR-21 and miR-22 are higher expressed in LSCs than in HSCs (Figure 3) [40].

Mir551b is the top differentially expressed miRNA between residual HSCs and LSCs, showing high expression in HSCs [40]. MiR-551b is not only highly expressed in residual HSCs in AML but also in HSCs residing in healthy bone marrow [41], suggesting a link between “stemness” and presence of miR-551b. Not only genuine normal stem cells have high mir-551b expression but also AML cases with an undifferentiated stem cell-like phenotype have [41]. Importantly, AML cases with enhanced miR-551b expression are associated with a poorer clinical outcome than those with lower miR-551b expression [41], potentially reflecting the influence of “stemness” on therapy sensitivity. Indeed, in ovarian cancer the expression of miR-551b is enhanced in the side population, the cell population that is enriched for cancer stem cells [132]. Mir-551b is located at the chromosome 3q26 locus, which is translocated and leading to overexpression of EVI-1 in a subset of AML patients. In ovarian cancer, amplification of 3q26 leads to increased expression of miR-551b, subsequently contributing to apoptosis resistance and increased survival and proliferation of the cancer cells. The mechanisms whereby miR-551b increases proliferation in ovarian cancer cells is not by decreasing the levels of mRNA targets but by binding to the STAT3 promoter and by recruitment of RNA polymerase II and TWIST1 [133]. In ovarian cancer and lung cancer cell lines, enhanced miR551b expression showed to be linked with therapy resistance [132,134]. Other miRNAs highly expressed in residual HSCs and potentially associated with HSC functions are miR-29b and miR-125b [40]. In general, both miRNAs are downregulated in AML patients as compared to HSCs [50,56]. However, it might be possible that miR-29b and miR-125b are higher expressed in a small subset of AML cases or in a small subpopulation of leukemic cells within the AML bulk. The research group of Marcucci supported a tumor suppressor role for miR-29b and used synthetic anti-miR-29b oligonucleotides as a novel strategy to eliminate AML cells [135,136]. MiR-29b overexpression had similar effects as the hypomethylating agents 5-azacytidine and decitabine [56] and its downregulation has been linked to promotion of DNA hypermethylation in AML cells by directly targeting DNMT3A, DNMT3B and SP1 [137,138]. MiR-29b upregulation has also demonstrated to inhibit cell proliferation, promote myeloid differentiation and induce apoptosis [135]. Together these data suggested that the consequences of overexpression of miR-29b is cell-type specific and may depend on the differentiation and/or transformation state of the cancer cell. The effect of enhanced miR-29b on the quiescence state of LSCs and thereby their chemotherapy sensitivity and survival has not been extensively investigated yet.

In AML, miR-125b is strongly upregulated as compared to whole healthy bone marrow, particularly in patients with a t(2;11)(p21;q23) [139]. Enhanced expression of miR-125b in myelodysplastic syndrome (MDS) and in AML with a (2;11)(p21;q23) resulted in a differentiation arrest, indicating a connection between high miR-125b and an immature leukemic phenotype [139,140]. Moreover, miR-125b overexpression causes a myeloproliferative disorder that progressed to an aggressive form of AML within 3–4 months [50,58,65]. Mice transplanted with hematopoietic progenitors overexpressing miR-125b led to various types of leukemia, including B-cell acute lymphoblastic leukemia, T-cell acute lymphoblastic leukemia or a myeloproliferative neoplasm depending on the degree of miR-125b expression [65].

MiR-181b is one of the upregulated miRNAs in LSCs as compared to HSCs [40]. MiR-181b is part of a miRNA signature expressed in cytogenetically normal (CN) AML containing high-risk molecular characteristics (e.g., NPM1 negative, FLT3-ITD positive) and is associated with a good prognosis [141]. Moreover, a 15 miRNA signature, including miR-181b, showed an association with the presence of the CEBPα mutation, possibly partly explaining the good prognostic characteristics of AML with high expression of the miR-181 family [142]. The overexpression of miR-181b promotes apoptosis and inhibits the viability of *MLL*-rearranged AML cells [143].

Lastly, miR-21 and miR-221, are higher expressed in LSCs and AML blasts than in HSCs and healthy bone marrow cells [34,40,144]. MiR-221 showed to be a biomarker distinguishing AML from acute lymphoid leukemia (ALL) [34,37]. An association between “stemness”, DNMT3A expression and miR-221 has been shown in breast cancer [145]. MiR-21 and miR-221 are both higher expressed in the pancreatic cancer cells residing in the side population and modulation of miR-21 and miR-221 in initiating stem-like cells affects tumorigenesis, metastasis, and chemotherapy resistance in pancreatic cancer [146]. MiR-21 showed to be overexpressed in AML with a NPM1 mutation [147]. Inhibition of miR-21 in the myeloid cell lines HL60 and K562 reduced cell growth, induced apoptosis and a G1 cell cycle arrest [148]. Moreover, several studies showed that downregulation of miR-21 in myeloid leukemia cell lines increased the sensitivity to various chemotherapeutic agents [149,150,151], making targeting of miR-21 a potential successful selective approach to sensitize LSCs for chemotherapy.

## 5. MicroRNAs Differentially Expressed between LSCs and Leukemic Progenitors

MiRNAs that are functionally involved in chemotherapy resistance and/or leukemia-initiating potential might be differentially expressed between LSCs and the chemotherapy sensitive AML bulk. By comparing miRNA expression in purified LSCs (CD34+CD38−) and leukemic progenitors (CD34+CD38+) 12 differentially expressed miRNAs were identified (Figure 3) [40]. The top three lower expressed miRNAs in LSCs as compared to leukemic progenitors are miR-1274a, miR-886 and miR-1305. Although there is not much information on the function of miR-1274a, it is suggested to be derived from tRNA processing rather than being a miRNA [152]. In gastric cancer cells, miR-1274a has been described as an oncogene involved in cell proliferation and migration by targeting FOXO4 [153].

Also miR-886 (vtRNA2-1, pre-miR-886, or CBL3) showed not to be a miRNA but a newly identified non-coding RNA (ncRNA) [154] that acts as a tumor suppressor targeting Protein Kinase R (PKR) [155,156]. PKR is a sensor that recognizes viruses and induces apoptosis to eliminate infected cells. Therefore, the nc886 signaling pathway in cancer cells is suggested to function in sensing and eliminating pre-malignant cells, analogous to PKR’s role in cellular innate immunity [154]. The nc886 is transcribed by RNA polymerase III (Pol III) [157] and is the first case of a Pol III gene whose expression is silenced by CpG DNA hypermethylation in several types of cancer. Low expression of nc886 has been associated with poor prognosis in AML, low risk MDS and small cell lung cancer [158,159,160], suggesting that its lower expression in LSCs might also be involved in a decreased response to chemotherapy in these cells. Thus, increasing nc886 expression might be a strategy to enhance chemotherapy sensitivity.

MiR-1305 showed to have a change in expression during the cell cycle in embryonic stem cells. Downregulation of miR-1305 facilitates the maintenance of pluripotency and increased cell survival, while its overexpression induced differentiation of pluripotent stem cells, increased cell apoptosis and sped up G1/S transition [161].

**Figure 4:**
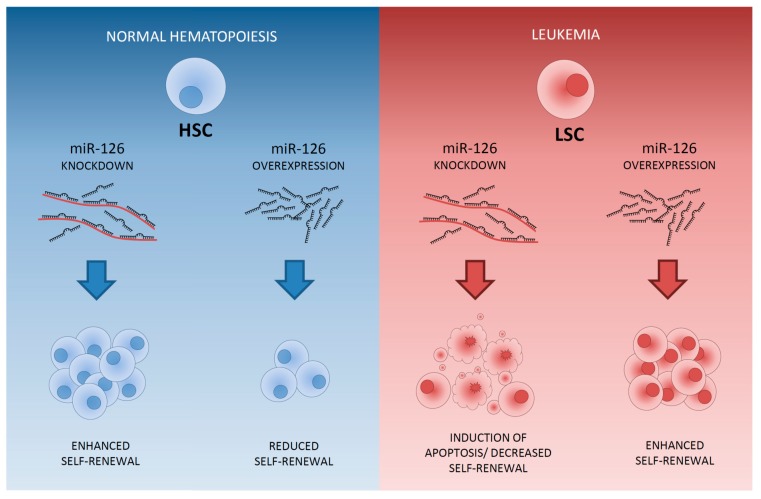
MiR-126 expression and modulation in normal hematopoiesis and leukemia. MiR-126 is highly expressed in normal HSCs. A reduction in miR-126 expression in HSCs, for example by lentiviral sponges, increases AKT signaling thereby inducing cell-cycle entry via CDK3 that leads to enhanced self-renewal and expansion of long-term HSCs. Overexpression of miR-126 in HSCs, by mimics or internal and external stimuli (cytokines), results in a reduction in AKT signaling impairing cell-cycle entry resulting in increased quiescence and a gradual loss of hematopoietic output. In leukemia, LSCs highly express miR-126 as compared to leukemic progenitors. Knockdown of miR-126 in LSCs increases AKT signaling, de-repressing CKD3, thereby inducing differentiation and proliferation leading to chemo-sensitivity and apoptosis. Overexpression of miR-126 in LSCs lowers AKT signaling and inhibits cell cycle entry leading to increased quiescence and self-renewal via the suppression of CDK3 resulting in chemotherapy resistance.

MiRNAs that are higher expressed in LSCs than in leukemic progenitors are miR-126-5p, miR-126-3p, miR-22, miR-335 and mir-150. MiR-126 is the top miRNA differentially expressed between CD34+CD38- LSCs and CD34+CD38+ leukemic progenitors. MiR-126 is high expressed in LSCs and even more enhanced in HSCs [40] (Figure 3). Moreover, miR-126 is part of an LSC-associated miRNA signature that was derived from functionally validated AML LSCs and its expression has been linked to LSC activity [60]. Patients with high miR-126 levels co-express genes that are present in stem cell gene signatures [19,40], implicating that miR-126 influences stem cell properties and maintenance of “stemness” in both normal and leukemic stem cells. In AML, high miR-126 expression is associated with poor survival and a high chance of relapse [40,60,162], reflecting the association of enhanced miR-126 activity with a decrease in chemotherapy sensitivity [60]. Both the overexpression and knockout of miR-126 result in enhanced leukemogenesis in cooperation with the t(8;21) fusion gene. MiR-126 overexpression has a stronger effect on long-term survival and progression of AML1-ETO9a-mediated LSCs in mice than does miR-126 knock-out [163]. Knockdown of miR-126 led to differentiation, apoptosis and reduction of AML growth [40,60] but also enhances responsiveness to chemotherapy [163]. Most importantly, targeting of miR-126 specifically reduced the clonogenic capacity of LSCs and leukemic progenitors without affecting normal HSCs [40,60] (Figure 4). Moreover, miR-126 targeting using nanoparticles resulted in the depletion of LSCs in an AML xenotransplantation model [162]. This specific effect of miR-126 is due to the opposite function of miR-126 in LSCs and in HSCs [40,60]. In LSCs, decreased miR-126 attenuates LSC quiescence and overrules chemotherapy resistance [60]. In HSCs, knockdown of miR-126 results in enhanced hematopoietic stem/progenitor cell proliferation, increases cell cycle progression and increases the number of stem cells (CD34+CD38-CD90+CD45RA-) [72] (Figure 4). MiR-126 targets multiple genes affecting the PI3K/AKT/mTOR pathway [60,72] and its discriminative function in LSCs and HSCs might reflect the opposite function of the PI3K/PTEN signaling pathway in LSCs and HSCs [164]. The distinct function of miR-126 in HSCs and LSCs makes it an ideal therapeutic target for depletion of LSCs without harming HSCs and potentially even enhancing hematopoietic recovery.

MiR-22 is upregulated in myelodysplastic syndrome (MDS) and leukemia and its aberrant expression correlates with poor survival [85]. MiR-22 was identified as being higher in LSCs than in leukemic progenitors but decreased in residual HSCs as compared to LSCs [40]. In contrast to these results, Jiang et al., showed that AML cells have lower miR-22 than CD34+ normal healthy bone marrow cells [104]. Mice that conditionally express miR-22 in the hematopoietic cell compartment showed decreased levels of 5-hydroxymethylcytosine (5-hmC), enhanced HSC self-renewal and developed MDS and hematological malignancies [85], indicating the oncogenic function of miR-22. MiR-22 targets TET2, a member of the TET methylcytosine dioxygenase family, and ectopic expression of TET2 suppressed the miR-22-induced phenotypes [85]. In contrast to this study, Jiang et al. [104] showed that miR-22 also has a tumor suppressive function. Forced expression of miR-22 inhibited leukemia development and maintenance in a xenograft AML mouse model [104]. Enhanced miR-22 expression, regulated by PU.1, resulted in monocyte/macrophage differentiation. In AML, there is downregulation of PU.1 and Mir-22 as well as upregulation of EVI-1. Reintroduction of miR-22, and the associated downregulation of its target EVI-1, relieved the differentiation block and inhibited the growth of AML bone marrow blasts [105].

## 6. Therapeutic Approaches to Specifically Eliminate LSCs: Sensitization to Chemotherapy

As miRNAs can regulate many genes and are also controlled by more than one gene, miRNAs are very appealing targets for specific anti-LSC therapies. Depending on the expression and function of miRNAs in AML, there are two approaches for developing miRNA-based therapies: antagonists and mimics. Antagonists can inhibit miRNAs and are usually single-stranded oligonucleotides [165]. Efficient silencing of miRNA activity in vivo requires the chemical modification of these oligonucleotides to improve their binding affinity, bio stability and pharmacokinetic properties. The most common modifications to increase the duplex melting temperature and to improve their resistance to nucleases include 2′-O-methyl- (2′-O-Me-), 2′-methoxyethyl- (2′-MOE-), 2′-fluoro- and the bicyclic locked nucleic acid (LNA) modifications [166,167,168]. Among these modifications, LNA exhibits the highest affinity toward complementary RNA [169]. Moreover, increased nuclease resistance is also achieved by substituting the phosphodiester (PO) backbone linkages with phosphorothioate (PS) which, apart from nuclease resistance, enhances the binding to plasma proteins leading to reduced clearance by glomerular filtration and urinary excretion. Moreover, inhibition of miRNAs by ultra-short 8-mer LNAs, which enable antagonism of a complete miRNA family, can result in therapeutic benefit in mouse disease models [170,171]. Currently, the most advanced therapeutic miRNA antagonists are directed against miR-122. One anti-miR-122 LNA has already been successfully tested in Phase II clinical trials for patients with hepatitis C [172]. Moreover a phase I clinical trial using a synthetic microRNA antagonist of microRNA-155 (MRG-106) is conducted in patients suffering from cutaneous T-cell lymphoma (CTCL).

Mimics, double stranded oligonucleotides, are used to restore miRNA function and have been designed to restore the function of various tumor-suppressive miRNAs [173]. For example miR-29b is known to be downregulated in AML [135] and a nanoparticle-based delivery system of miR-29b mimics in AML blasts showed decreased AML cell growth and impaired colony formation in a mice model of AML [136]. The use of lenti-, adeno- or adeno-associated viruses to drive the expression of a miRNA has been successfully applied to reduce tumor growth in mouse models [174,175]. The clinical application of a miRNA mimic of miR-34 (MRX34) is at the moment tested in a phase-I trial including patients with hematological malignancies [176].

The specific expression of miRNAs during haematopoiesis can also be used to specifically express a gene in differentiated hematopoietic cells. In a lysosomal storage disorder, there is a need for specific expression of the enzyme galactocerebrosidase in differentiated hematopoietic cells since its expression in HSCs results in toxicity. The enhanced expression of miR-126 in HSCs can inhibit the expression of galactocerebrosidase from a lentiviral construct containing miR-126 binding sites in HSCs, while miR-126 expression decreases during myeloid differentiation thereby causing expression of galactocerebrosidase and restoring lysosomal storage [59].

MiRNA-based therapy might not only function as a single agent but also holds great potential in complementing currently used chemotherapeutics. Since a single miRNA can induce global changes in overall gene expression, modulation of miRNA expression might be very effective in targeting a multi-factorial phenomenon like drug resistance. MiRNA modulation has been shown to have the capacity to enhance the response and suppress the resistance to cytotoxic therapies [177,178].

## 7. The Delivery of MiRNA Modulators to AML LSCs within the Leukemic Bone Marrow

Successful delivery of therapeutic miRNA(s) to the leukemic cells in the AML bone marrow, without inducing toxicity, is the final challenge. The charged miRNAs have a small size and low molecular weight making it possible to formulate them into effective delivery systems which reduce their clearance and degradation in the blood [179]. Examples of delivery systems for mimics and anti-miRs are lipids, polyethylenimine, dendrimers, poly (lactide-*co*-glycolide) particles but also naturally occurring polymers, such as chitosan, protamine and atelocollagen [136,179,180,181]. Importantly, the first liposome-formulated mimic is currently being tested in a Phase I clinical trial in patients with unresectable primary liver cancer. Beside the delivery of mimics and anti-miRs by formulation, viral constructs can be used [174,175].

Marcucci et al. developed a transferrin-conjugated nanoparticle delivery system conjugating transferrin (Tf) to PEGylated lipopolyplex nanoparticles (Tf-LPs) which incorporates protamine as a DNA condensing agent, pH-sensitive fusogenic lipids to improve cytoplasmic delivery, and Tf as the targeting ligand specific for cellular delivery (commonly overexpressed on cancer cells and also in AML) [182]. In a study that tested the delivery of miR-29b loaded transferrin-conjugated nanoparticles (Tf-NP-miR-29b) to leukemic cells in a xenograft mouse model, high uptake and strong downregulation of miR-29b targets in the leukemic cells was observed [136]. Tf-NP-miR-29b suppressed AML growth, impaired colony formation, and reduced cell viability in AML patient samples. In addition, Tf-NP-miR-29b also reduced spleen weight and increased overall survival in NSG mice transplanted with AML cell lines [136]. Next to miR-29b, transferrin conjugated nanoparticles containing miR-126 have recently been used. As previously mentioned, treatment with Tf-NP-miR-126 specifically targets the LSC leading to diminished engraftment of both human and mouse AML in secondary recipient transplantations [162]. Before these works, in vivo targeting of miR-196b was also reported to successfully eradicate LSCs from AML blasts harboring MLL translocations [183]. A mimic Tf-NP-miR181a treatment downregulated KRAS, NRAS and MAPK1 and decreased AML growth in mice, resulting in a longer survival compared to the controls [184]. The use of nanoparticles to force expression of miR-22, which is often downregulated in AML, significantly inhibited AML progression in vivo [104]. Altogether, these studies show the great potential for future miRNA based treatment and the use of nanoparticles to deliver them to AML cells.

## 8. Concluding Remarks

Two decades ago miRNA research started with the expression profiling of various hematopoietic cell populations and types of leukemia which provided us with an enormous number of miRNAs that could potentially play a regulatory role in normal and malignant hematopoiesis. Indeed, many of these miRNAs now have established involvement in controlling differentiation, apoptosis, proliferation and self-renewal in hematopoiesis and leukemia. The most extensively studied miRNAs include miR-125b, miR-29b and miR-126 which are all involved in stem cell regulation and leukemogenesis. MiRNA-based therapy that modulates these miRNAs to prevent leukemogenesis or treat frank leukemia is now possible and holds great potential. Although recent reports on in vivo miRNA treatment are promising, still many issues in optimizing delivery methods and unknown factors like toxicity, due to off-target effects, should be evaluated and solved. It is therefore wanted that future miRNA research focusses on the efficient in vivo delivery and specific targeting of leukemia (stem cells) to really bring miRNAs from bench to bedside.

## Figures and Tables

**Table 1:** miRNAs involved in myelopoiesis which are involved in key (stem) cell processes (differentiation, self-renewal, apoptosis and proliferation) and their identified targets.

Cell Stage	microRNA	Target	Function		References
**HSC**	Let-7	Hmg2a	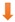	self-renewal	[61]
	miR-12	Tip110	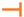	differentiation	[62]
	miR-125a	BAK1	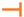	apoptosis	[30,31,58]
	miR-125b	ABTB1/CDC25C/PPP1CA	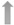	proliferation	[63,64]
		Bmf/KLF13/p53	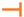	apoptosis	[56,58,65]
		STAT3/c-JUN/JUND/LIN28A/CBFB	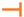	differentiation	[63,66,67,68,69,70]
	miR-126	HOXA9/PI3K/AKT2/CRKII	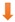	self-renewal	[71,72]
	miR-132	FOXO3	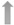	proliferation	[73]
	miR-146a	TRAF6/IRAK1/STAT1	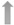	self-renewal	[74,75,76]
	miR-17-92 cluster	E2F1/E2F2	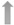	proliferation and block differentiation	[77,78]
		PTEN/Bim	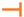	apoptosis	[79,80]
	miR-196b	HOXA9/MEIS1/FAS/HOXB8	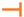	differentiation	[81,82]
	miR-24	Bim/CASP9	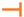	apoptosis	[83]
	miR-29a	Dnmt3a	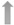	self-renewal	[57]
	miR-33	p53	self-renewal		[84]
	miR-22	Tet2	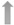	self-renewal	[85]
**MPP**	miR-17/20/93/106	SQSTM1	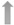	differentiation towards myeloid progenitors	[54,86]
	miR-24	Unknown	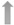	differentiation towards myeloid progenitors	[83]
	miR-29a	HBP1, FZD5, TPM1	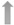	differentiation towards myeloid progenitors	[55]
	miR-520h	ABCG2	differentiation towards myeloid progenitors		[49]
	miR-181a	Bcl2, CD69	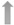	differentiation towards lymphoid progenitors	[87,88,89]
**CMP**	miR-142-3p	CCNT2/TAB2	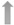	granulocytic-macrophage differentiation	[90]
	miR-155	PU.1	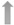	granulocytic-macrophage differentiation	[91]
	miR-29a	CCNT2/CDK6	granulocytic-macrophage differentiation		[90]
	miR-130a	C/EBPɛ	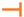	granulocytic differentiation	[92]
**GMP**	miR-17-5p/20a/106a	RUNX1	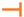	monocytic differentiation and maturation	[93]
	miR-223	MEF2C	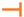	progenitor proliferation and granulocyte differentiation	[94]
	miR-223 miR-27	NFI-A/E2F1	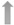	granulocytic differentiation	[95,96,97]
		RUNX1	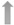	granulocytic differentiation	[98]
	miR-30c	NOTCH1	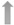	granulocytic differentiation	[99]
	miR-34a	E2F3	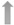	granulocytic differentiation	[100]
	miR-424	NFI-A	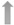	monocytic differentiation	[101]
	miR-486-3p	MAF	Skews from monocytopoiesis towards granulopoiesis		[102]
	miR-105	MYB	megakaryopoiesis		[103]
	miR-22	PU.1, MECOM	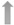	monocytic differentiation	[104,105]
	miR-181a	Unknown	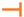	megakaryocytic differentiation	[106]
		Lin28, let7	megakaryocytic differentiation		[107]
**MEP**	miR-125b	Unknown	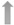	proliferation and self-renewal	[108]
	miR-126	MYB	Skews from erythropoiesis towards megakaryopoiesis		[109]
	miR-145	Fli-1	Skews from megakaryopoiesis towards erythropoiesis		[110]
	miR-146a	CXCR4	Impairs megakaryocytic proliferation, differentiation and maturation		[111]
	miR-15	MYB	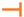	erythropoiesis	[112]
	miR-150	MYB	Skews from erythropoiesis towards megakaryopoiesis		[109,113,114]
	miR-155	ETS-1/MEIS1	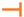	megakaryocytic proliferation and differentiation	[115]
	miR-199b-5p	c-Kit	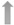	erythroid differentiation	[116]
	miR-221/222	c-Kit	Impairs proliferation and accelerates differentiation of erythroid cells		[32]
	miR-223	LMO2	Skews from erythroid towards megakaryocytic differentiation		[117]
	miR-23	SHP2	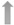	erythroid differentiation	[118]
	miR-27a/24	GATA2	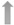	erythroid differentiation	[119]
	miR-299-5p	Unknown	Skews from erythroid-monocytic towards megakaryocytic-granulocytic differentiation		[120]
	miR-34a	MYB/CDK4/CDK6	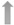	megakaryocytic differentiation and inhibit cell cycle	[121]
	miR-376a	CDK2	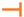	erythroid differentiation	[122]
	miR-451/144	GATA2	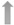	erythroid differentiation	[123,124,125]
	miR-486-3p	MAF/BCL11A	Skews from megakaryopoiesis towards erythropoiesis		[102,126]
	miR-146b	PDGFRA	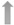	erythrocytic-megakaryocytic differentiation	[127]
